# Nematode epibionts on skin of the Florida manatee, *Trichechus manatus latirostris*

**DOI:** 10.1038/s41598-020-79879-7

**Published:** 2021-01-13

**Authors:** Rafael Gonzalez, Natsumi Kanzaki, Cathy Beck, William H. Kern, Robin M. Giblin-Davis

**Affiliations:** 1 Fort Lauderdale Research and Education Center, Department of Entomology and Nematology, University of Florida/IFAS, 3205 College Avenue, Davie, FL 33314-7799 USA; 2 Kansai Research Center, Forestry and Forest Products Research Institute, 68 Nagaikyutaroh, Fushimi, Kyoto, 612-0855 Japan; 3 Wetland and Aquatic Research Center, United States Geologic Survey, 7920 NW 71st Street, Gainesville, FL 32653 USA

**Keywords:** Biological techniques, Ecology, Evolution, Genetics, Microbiology, Molecular biology, Systems biology, Zoology, Ecology, Environmental sciences, Ocean sciences

## Abstract

A survey for the presence of nematodes on the skin of the native Florida manatee, *Trichechus manatus latirostris* from Crystal River, Florida was conducted during annual manatee health assessments. A putative isolate of *Cutidiplogaster manati* (Diplogastridae) and two other nematodes belonging to the same family were recovered from mid-dorsal tail skin-scrapings from all sampled winter-collected healthy wild adult manatees during two successive years (2018–2019). Qualitative abundance estimates of these three species of diplogastrid nematodes suggest that an average wild Florida manatee adult might possess between 30,000 and 120,000 nematodes on its tail dorsum and that the entire body dorsum including the tail might possess 160,000–640,000 nematodes in roughly equal ratios. Attempts to culture these nematodes on a variety of different culture media were unsuccessful but examination of the mouth (stomatal) morphology suggests specialized feeding on microbes such as diatoms or predation on other nematodes. No skin lesions were observed during the 2018–2019 samplings suggesting that under normal conditions these nematodes are highly specialized free-living epibionts of the skin that are tightly bound to this niche and horizontally transferred between individual manatees in an analogous fashion to human skin mites (*Demodex folliculorum* and *D. brevis*). Molecular phylogenetic inferences using sequences of near full length SSU and D2–D3 expansion segments of LSU rRNA genes revealed a putative new morphospecies in *Cutidiplogaster* sister to *C. manati* that was monophyletic with several named *Mononchoides* species, and another putative new morphospecies that formed a clade with several undescribed species similar in appearance to *Mononchoides* as well as *Tylopharyx*, *Eudiplogasterium*, *Paroigolaimella* and *Sachsia*.

## Introduction

Nathan A. Cobb, the father of American nematology, famously wrote in 1914, “…if all the matter in the universe except the nematodes were swept away, our world would still be dimly recognizable… The location of the various plants and animals would still be decipherable, and, had we sufficient knowledge, in many cases even their species could be determined by an examination of their erstwhile nematode parasites"^1^. Over the past 100 years, this prediction about the diversity and abundance of nematode roundworms has been supported by numerous studies and several nematode model systems have emerged for studying comparative and evolutionary biology^2–4^. In this spirit, what would happen if you looked closely at the skin of a manatee? Would Cobb’s prophecy ring true? Would you find nematode(s) specifically adapted for riding, living, feeding, and reproducing on manatee skin through the different environmental challenges that they face? Cobb’s prediction was more about the traditional internal nematode parasites of vertebrates and invertebrates, which are known from manatees^5^, but recent observations^6,7^ suggest that nematodes can be recovered from their skin which begged the preceding questions for further study.

Nematodes of the Diplogastridae are often found symbiotically associated with terrestrial insects/invertebrates and usually affiliated with wet/moist niches involving dead or decomposing substrates and their associated organisms^8^. The occurrence of diplogastrids from marine/brackish water environments is rare^9^, i.e., there are only two reports, namely, *Allodiplogaster* (= *Eudiplogaster*) *paramatus* (Schneider, 1938)^10^, a diatom-eating worm from brackish water in mud flats in the southeast of the Ems-Dollard estuary in The Netherlands^11^ and *Cutidiplogaster manati*^6^ from skin samples taken from captive West Indian manatees (*Trichechus manatus manatus*) located in an aquarium in Okinawa, Japan^6^. Thus, the body surface of the manatee is of special interest because members of this nematode family are rarely found in environments with widely ranging salinities (euryhaline) or associated with vertebrates, except in the recent case of *C. manati* which was only cursorily examined for its relationship involving captive manatees far from their natural distribution^6^. In addition, molecular data provided in the original description of *C. manati* was insufficient for inferring its placement in a modern molecular phylogenetic framework involving other sequenced members of the Diplogastridae.

Beck and Forrester^5^ reviewed the literature for all helminth parasites of the sirenians of the world, as well as provided the first quantitative report of helminths from the Florida manatee (*T. m. latirostris*). The only nematode recovered from the Florida manatee which ranges from freshwater to marine habitats was the ascarid, *Heterocheilus tunicatus* Diesing from the mucosa and lumen of the stomach and small intestines. This nematode has also been reported from the stomach/small intestines of the Amazonian manatee (*T. inunguis* Natterer) which is restricted to freshwater habitats in the Amazon River Basin. There had been no reports of external nematode parasites involving the skin of sirenians^5^. However, unidentified nematodes were observed among the micro- and meso-flora/fauna of biofouling communities on the skins of about 60–70% of both captive and winter-collected free-ranging wild Florida manatees^7^. In that study, papillomavirus-induced skin lesions were observed in the captive population of Florida manatees from Homosassa Springs, FL, but not the wild populations surveyed from Tampa and Naples, FL. That nematodes were observed in similar association rates between the captive (with lesions) and free-ranging (without lesions) populations suggests that although nematodes appear to comprise a potentially predictable component of the Florida manatee skin biofouling layer, a causal relationship between nematodes and skin disease has not been established^7^. Unfortunately, because the nematodes were not identified beyond the phylum level, it is impossible to know whether there might be a predictable epibiont/basibiont relationship between certain species of nematodes and manatees^7^.

In the present study, the nematode fauna from the body surfaces of winter-surveyed wild native Florida manatees (*T. m. latirostris*) was examined over several years in an attempt to re-isolate *C. manati* and obtain its molecular profile for placement in a modern molecular phylogenetic framework, and to better understand its relationship with its vertebrate host. In the process, two additional diplogastrids new to science were discovered as cohabiting Florida manatee epibionts that are further described and discussed herein.

## Results

**Figure 1:**
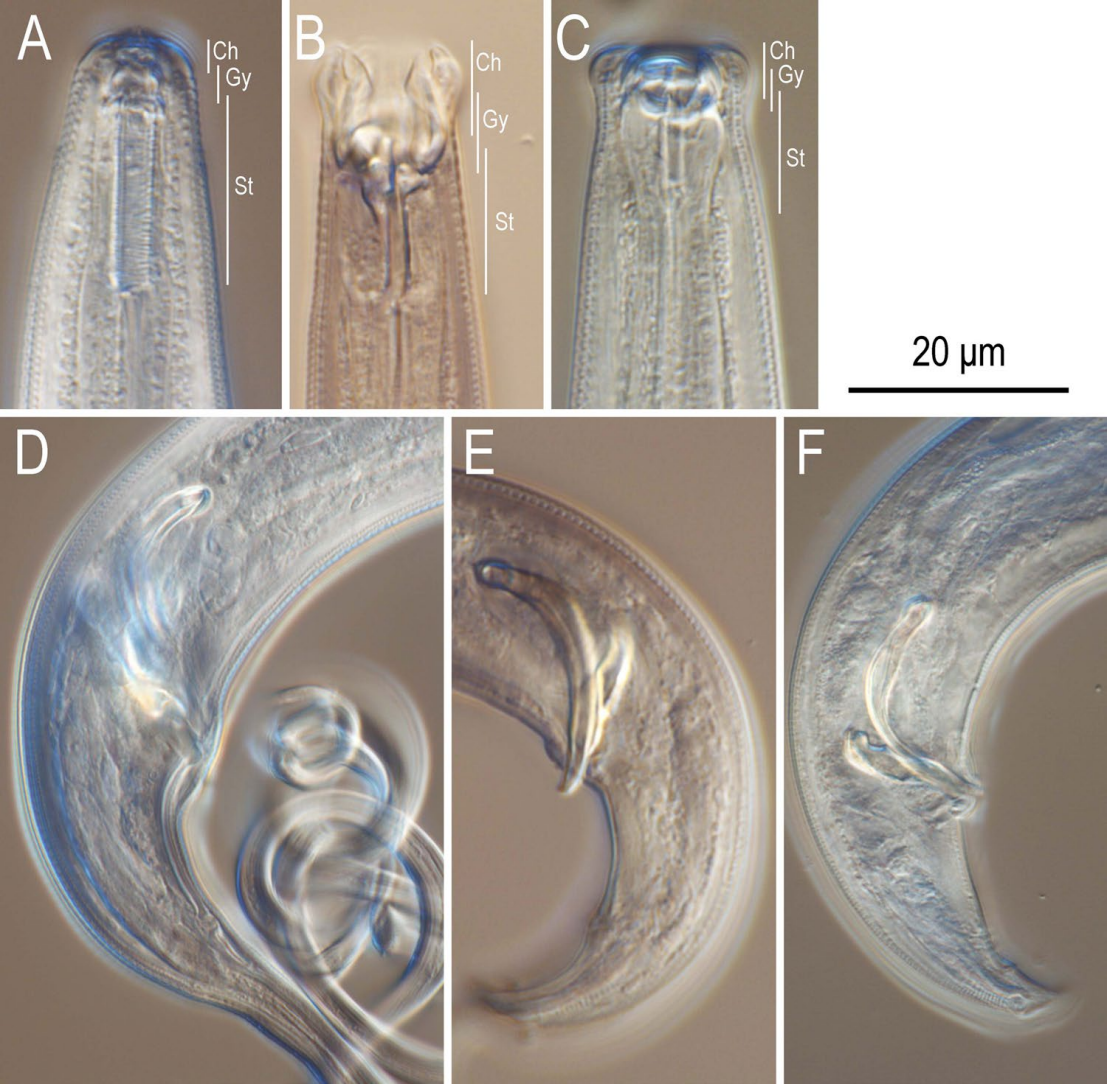
Anterior and male tail regions of three manatee-associated nematodes. (**A**–**C**) Stomatal region of *Cutidiplogaster manati* (**A**), “LT” (**B**) and “ST” (**C**); (**D**–**F**) Male tail region of *Cutidiplogaster manati* (**D**), “LT” (**E**) and “ST” (**F**). Cheilostom (Ch), Gymnostom (Gy) and Stegostom (St) are suggested with lines.

**Figure 2:**
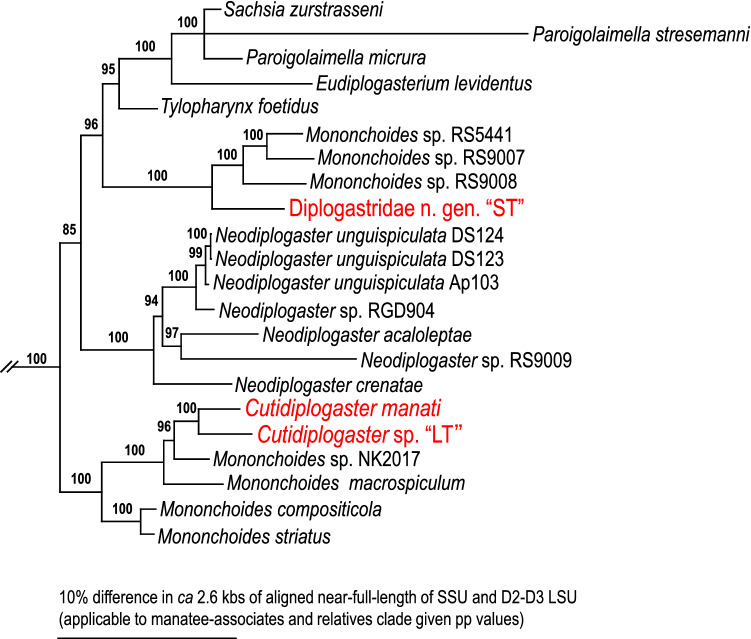
The phylogenetic status of three manatee-associated nematodes among closely-related species. The combined Bayesian tree was inferred from near full length of SSU and D2-D3 LSU under GTR + G + I model applied for both loci. The parameters are as follows: lnL = − 24,768.25718; freqA = 0.25; freqC = 0.21; freqG = 0.27; freqT = 0.27; R(a) = 1.00; R(b) = 2.88; R(c) = 2.38; R(d) = 0.88; R(e) = 4.25; R(f) = 1.00; Pinva = 0.38; Shape = 0.57) for SSU, and lnL = − 27,032.96896; freqA = 0.21; freqC = 0.22; freqG = 0.32; freqT = 0.25; R(a) = 0.43; R(b) = 1.50; R(c) = 0.79; R(d) = 0.36; R(e) = 3.07; R(f) = 1.00; Pinva = 0.20; Shape = 1.03). Posterior probability values exceeding 50% are given on appropriate clades. The full phylogenetic tree is provided in Supplementary Fig. S1.

*Cutidiplogaster manati* (Fig. 1, Suppl. Figs. S2–S4) was isolated from all wild sampled Florida manatees from Crystal River, Florida from 2018–2019 (Tables 1 and 2). In addition, two distinct diplogastrid nematode morphospecies were also identified from the dorsal mid-tail samplings (Tables 1 and 2). One of these nematode morphospecies has a somewhat long tail (Fig. 1, Suppl. Figs. S5–S7), but not quite as long as *C. manati* (Suppl. Figs. S3, S4, S6, and S7). The other new diplogastrid morphospecies has a very short tail (Fig. 1, Suppl. Figs. S8, S9). These diplogastrid morphospecies were referred to as Long Tail (LT) and Short Tail (ST) and superficially resembled a *Tylopharynx* or *Mononchoides* in terms of their mouthparts. The mouthparts of members of the nematode family Diplogastridae are usually separated into three elements from the anterior, i.e., a short tube-shaped cheilostom which is often composed of plates or a simple ring, a short or long tube-shaped gymnostom without any special armature, and a relatively shallow cup-shaped stegostom with teeth or denticles (Fig. 1). All three manatee nematodes have deep and well-developed stegostomatal elements with various and uniquely shaped teeth (Fig. 1). The LT and ST diplogastrid nematode morphospecies appeared morphologically closer to each other relative to stomatal morphology than to *C. manati*, although one (i.e., LT) does have a long tail like *C. manati*. Molecular phylogenetic inferences (Fig. 2) revealed that the LT diplogastrid morphospecies is monophyletic with *C. manati* and apparently congeneric with it and three sequenced and two nominal *Mononchoides* species. These are *Mononchoides* sp. NK-2017 isolated from the palmetto weevil *Rhynchophorus cruentatus*^12^, *M. macrospiculatum* isolated from the red palm weevil *R. ferrugineus*^13^, *M. compositicola*^14^ and *M. striatus*. The LT diplogastrid morphospecies is clearly a putative new species closest to or sister with *C. manati* that shares highly derived stomatal morphology and an association with the Florida manatee but should probably be reconsidered as a highly derived *Mononchoides,* not part of a separate genus (*Cutidiplogaster*) (Fig. 2). The ST diplogastrid morphospecies is also a putative new genus/species that is monophyletic with three sequenced “*Mononchoides*” species in GenBank^15,16^ from scarabaeoid beetles (RS5441, RS9007 and RS9008) that are more closely related to *Tylopharynx, Eudiplogasterium, Paroigolaimella* and *Sachsia* than named *Mononchoides* (see Fig. 2).

**Table 1:** Skin sampling results and relative abundance of nematodes on the Florida manatee from Crystal River, Florida during the 2018 health assessments.

USGS manatee ID	Sample plate	*Cutidiplogaster manati*	LT	ST	Visible skin remnants
CCR18-19	Sample A	+++	+++	+++	P
	Sample B	+++	+++	+++	P
	Sample C	0	+++	0	P
	Sample D	+++	+++	+++	P
	Sample E	0	+++	+++	P
	Sample F	+++	+++	0	P
CCR18-20	Sample A-1	0	+++	0	A
	Sample B-1	+++	+++	+++	A
	Sample C-1	0	+++	0	A
	Sample D-1	0	+++	+++	A
	Resample A-2	0	+++	0	P
	Resample B-2	0	+++	0	P
	Resample C-2	0	+++	0	P
	Resample D-2	+++	+++	0	P
CCR18-21	Sample A	0	0	0	A
	Sample B	0	+	0	P
	Sample C	0	++	++	A
	Sample D	++	++	++	A
	Sample E	++	++	++	P
	Sample F	++	++	++	P
CCR18-22	Sample A	0	0	0	A
	Sample B	+	+	0	A
	Sample C	0	++	++	P
	Sample D	0	++	++	A
	Sample E	++	++	++	P
	Sample F	++	++	++	A
CCR18-23	Sample A	+++	+++	+++	A
	Sample B	+++	+++	+++	A
	Sample C	0	+++	+++	A
	Sample D	0	+++	+++	A
	Sample E	+++	+++	+++	A
CCR18-24	Sample A	+++	+++	+++	A
	Sample B	+++	+++	+++	A
	Sample C	+++	+++	+++	A
	Sample D	+++	+++	+++	A
	Sample E	+++	+++	+++	A
	Sample F	+++	+++	+++	A
CCR18-25	Sample A	+++	+++	+++	P
	Sample B	+++	+++	+++	P
	Sample C	+++	+++	+++	P
	Sample D	+++	0	+++	P
	Sample E	+++	+++	+++	P
	Sample F	+++	+++	+++	P
	Sample G	+++	+++	+++	P
	Sample H	+++	+++	+++	P
	Sample I	+++	+++	+++	P

Estimated measurement of abundance was recorded for each morphospecies observed on each sample plate. This was categorized and recorded as 0 = absent, +  = 1–15 nematodes visible,++ = 15–99 nematodes visible, and +++  = 100 or more nematodes visible. Visible skin remnants were categorized as A = absent, or P = present. On manatee CCR18-20, sampling first used minimal pressure (samples A-1 to D-1) and then each location was resampled with increased pressure (samples A-2 to D-2). For all other sampled manatees, each different sample plate represents a pseudoreplicate sample taken from a different arbitrary location near the mid-tail dorsal surface. Samples labelled under a specific manatee ID with a different letter represent a different 5 × 5 cm mid-dorsal tail sampling on the same manatee.

**Table 2:** Skin sampling results and relative abundance of nematodes on the Florida manatee from Crystal River, Florida during the 2019 health assessments.

USGS manatee ID	Sample plate	*Cutidiplogaster manati*	LT	ST	Visible skin remnants
CCR19-01	Sample A	+++	+++	+++	A
	resample A-1	+++	0	+++	P
	resample A-2	+++	+++	+++	P
	resample A-3	+++	+++	+++	P
CCR19-02	Sample A	+++	+++	+++	P
	resample A-1	+++	+++	+++	P
	resample A-2	+++	+++	+++	P
	resample A-3	++	++	++	P
	Sample B	+++	+++	+++	P
	Sample C	+++	+++	+++	P
	Sample D	+++	+++	+++	P
CCR19-03	Sample A	0	0	0	P
	Sample B	++	++	0	P
	Sample C	0	+	0	P
	Sample D	0	0	0	P
CCR19-04	Sample A	0	0	+	A
	resample A-1	++	++	++	P
	resample A-2	++	++	++	P
	resample A-3	++	++	++	P
CCR19-05	Sample A	0	++	++	P
	resample A-1	0	++	++	P
	resample A-2	++	++	++	P
	resample A-3	++	++	++	P
CCR19-06	Sample A	++	++	0	A
	resample A-1	0	++	0	P
	resample A-2	++	0	0	P
	resample A-3	++	++	++	P
CCR19-07	Sample A	0	+	+	P
	resample A-1	+++	+++	+++	P
	resample A-2	+++	+++	+++	P
	resample A-3	+++	+++	+++	P

Estimated measurement of abundance was recorded for each morphospecies observed on each sample plate and categorized as 0 = absent, +  = 1–15 nematodes visible,++ = 15–99 nematodes visible, and +++  = 100 or more nematodes visible. Visible skin remnants were categorized as A = absent, or P = present. All manatees except CCR19-03 were sequentially sampled using increasing pressure. Sequential sampling first used minimal pressure (sample A) and then each location was resampled with increased pressure (resamples A-1 to A-3). On manatee CCR19-03, each different sample represents a pseudoreplicate taken from a different arbitrary location near the mid-tail dorsal surface. On manatee CCR19-02, three additional pseudoreplicate sample were also taken from different arbitrary location near the mid-tail dorsal surface.

Nematode population surveys (presence, absence, relative abundance of different morphospecies) from the collected samples were observed on the freshly collected plates under magnification and an abundance estimate for each morphospecies in the sample was made. These were recorded for 2018 (Table 1) and 2019 (Table 2). All three diplogastrid nematode morphospecies, including *C. manati*, were identified in samples from every manatee sampled during 2018, but not for every sample. For the single manatee that was sequentially sampled with light and then increased pressure, i.e., CCR18-20, there was no apparent difference between which nematodes were recovered. However, *C. manati* was located in the second of four sequential samples and only one of four separate samples that were collected.

A general estimate of nematode abundance for the dorsal mid-tail surface area per manatee adult was done by converting the abundance category data in Table 1 to: 0 = 0, +  = 10, ++  = 50 and +++  = 100 nematodes per each morphospecies for every non-sequential sample to calculate an average number of nematodes per adult per cm^2^. Based on these calculations, the average number of *C. manati*, LT morphospecies and ST morphospecies was 2.26, 3.19, and 2.76 nematodes per cm^2^, or 27%, 39% and 34% of the nematode skin fauna. To generate an estimate of the average dorsal surface area of the mid-tail alone versus the dorsal body plus the mid-tail of an average Florida manatee adult, we used the curvilinear total length and umbilicus girth circumference (circumference at the level of the manatee’s navel) data from seven female and six male adult manatees reported in Table S1. The umbilicus girth measurement was used to calculate the radius for the circular area estimating the dorsal tail area and the radius “a” for an ellipse calculation that used ½ total curvilinear body length as radius “b” when estimating the dorsal body area of each manatee. This produced mean length and area estimates of 262/310 cm, 64/66/cm, 15,320/16,136 cm^2^, 3730/3435 cm^2^, and 19,050/19,571 cm^2^ for mean respective female/male total curvilinear length, tail length, body only area, tail area, and total dorsal body plus tail area. Thus, an average adult female/male tail dorsum possessed 30,636/28,211 nematodes and an average adult female/male dorsum possessed 156,452/160,727 nematodes in the previously stated percentages.

In 2019, all three diplogastrid nematode morphospecies were found on every sampled adult manatee and on every sequentially sampled area (Table 2). When *C. manati* was not observed on every sequential sample from each sampled area during 2019, it was always absent from the biofilm layer but present when dead skin was collected. This was true for manatees CCR19-04, CCR19-05 and CCR19-07 (Table 2). This suggests that *C. manati* preferred a greater depth within the dead skin matrix. However, for CCR19-01 and CCR19-02, a high relative abundance of all three diplogastrid nematode morphospecies was found at every sampling level. One juvenile manatee, CCR19-03, was sampled and sequential sampling was not performed on this individual because the dead skin layer was removed too easily and it was judged that increased pressure might result in injuring the manatee’s epidermis (Table 2). In addition, nematode abundance was relatively sparse and the ST diplogastrid morphospecies was not detected among four samples (Table 2). Based on calculations from relative abundance data from Table 2, the average number of *C. manati*, LT morphospecies and ST morphospecies was 2.64, 2.65, and 2.65 nematodes per cm^2^, or 33%, 34% and 33% of the nematode skin fauna and very similar to results from 2018. However, the more extensive resampling of the same 25 cm^2^ area suggests that the same area could generate about 3–4X more nematodes with continued deeper sampling of the skin. Thus, an average adult manatee could have 90,000–120,000 nematodes on the tail dorsum and the body and tail could possess more than 480,000–640,000 nematodes.

## Discussion

Ultimately, Cobb’s prophecy holds true for the Florida manatee. If everything in the world was swept away except the nematodes, the location of Florida manatees would still be decipherable as apparitions of ghostlike sea cows created by the communities of specialized diplogastrid nematode skin epibionts reported in this study. Three different diplogastrid nematode morphospecies were identified and consistently recovered in dorsal skin scrapings from healthy wild adult Florida manatees. Based upon the collection data and observed morphology of these Florida manatee roundworms they have clear and specific adaptations for being epibionts associated with the skin of their hosts through the different environmental challenges that they face together. The exceptionally long tails of *C. manati* and the LT morphotype suggest mechanisms for anchoring and thriving in the epidermal substrate. That the same dorsal tail sampling area continued to produce similar levels of abundance for all three nematode morphospecies (see CCR-19-01 and 02 in Table 2) with multiple scrapings suggests that the nematode communities may possess some niche stratification associated with depth in the manatee skin and biofouling layer. The three different diplogastrid nematode morphospecies need to be further explored relative to abundance and distribution on the dorsum and venter of the manatee especially relative to the currently unknown feeding niches that each morphotype occupies.

Most Diplogastridae are predatory/free-living nematodes or insect associates with no known vertebrate parasitic members, making it less likely that they are causal agents of skin disease^,^^17^. However, we cannot rule out how they might negatively interact with manatees under stress. None of the manatees sampled (Tables 1 and 2) had skin lesions present, but all the adult manatees had all three diplogastrid nematode morphospecies present. In the original species description for *C. manati*^6^, the authors specified that the nematodes were anchored to epidermal bumps, whereas an ulcerative papillomavirus lesion like that on captive or immunosuppressed manatees^7,18^ lacks epidermal bumps since it disrupts the epidermis. While the authors did not specify the type of lesions found on the captive manatees with *C. manati* in Japan, there is no record of skin lesions in manatees which do not disrupt the epidermis. Other forms of lesions which are non-ulcerative, such as those induced by Cold Stress Syndrome or brevitoxicosis, also disrupt normal epidermal features because they manifest as warts or keratonic flat skin^18^. Papillomavirus can also manifest as similar non-ulcerative lesions on manatees, but still replace normal epidermal features^18^. Thus, it is possible that the *C. manati* collected from lesions by Fürst von Lieven et al.^6^ were from the border of healthy tissue surrounding the lesions or just contaminants and not the causative agent of the lesions.

The 100% skin association rate of the three diplogastrid nematode morphospecies and adults of the Florida manatee is analogous to the symbiotic relationship between human skin mites and humans (*Homo sapiens*). Human skin mites *Demodex* spp*.* live and reproduce within the hair follicles of terrestrial mammals and feed on dead skin cells^19^. They are considered ubiquitous symbionts of humans acquired by horizontal transmission^19^. The manatee diplogastrid nematodes could be acquired by juvenile manatees in a similar way from their mothers, and this was suggested by the sparse numbers on the young calf in 2019 (CCR19-03 in Table 2) which was the only individual sampled between 2018–2019 that did not have all three morphospecies present. In a recent study comparing the population genetics of the human skin mite *D. folliculorum*, it was reported that different human populations had skin mites that were genetically distinct from skin mites living on human populations living on other continents and could be separated into four distinct clades^19^. It was estimated that the last common ancestor shared by the four different mite clades occurred more than three million years ago. Since this is earlier than the first appearance of modern humans, the different mite species are hypothesized to have co-evolved with their ancestral human hosts^19^. While the ecology of these manatee-associated diplogastrid nematodes appears to be very different from that of human skin mites based upon the morphology of their mouths and their aquatic versus terrestrial life histories, there may be interesting parallels in their population genetics relative to their host manatee populations.

Although the morphology appears identical, *C. manati* needs to be collected again from populations of the West Indian Manatee, *T. m. manatus* (either from the aquarium in Japan or from its native range in eastern Mexico) to confirm that the species found on the Florida manatee, *T. m. latirostris* is genetically identical. This is necessary because the sequences from the original species description for *C. manati* isolated from a West Indian Manatee in an aquarium in Japan were suboptimal for inclusion in modern phylogenetic analyses and cannot be compared against the new sequence data generated for *C. manati* found on the Florida manatee. A comprehensive study comparing the population genetics of diplogastrid nematodes found on different manatee subspecies and species as well as dugongs from around the world could potentially reveal interesting patterns similar to those found in the skin mites associated with different human population groups. In addition to *T. manatus*, there are two other *Trichechus* species in the world, i.e., the Amazonian manatee, *T. inunguis* and the African manatee, *T. senegalensis* Link. It would be interesting to examine these two congeners to test the commonality of the origins of their nematode epibionts (if present), and the co-evolution and/or host switching patterns^20^.

Diplogastrid nematodes mostly occur in terrestrial and freshwater habitats. They are primarily found in biofouling-like environments, such as inside rotting palm trees^12^, or as halophytes in brackish wastewater conditions^11^. Diplogastrid nematodes are also associated with insects in terrestrial environments^21^, and this could provide a challenging osmotic environment, especially when the nematode is an associate of an insect but will also spend much of its life-cycle living inside, for example a fig fruit^22,23^. Therefore, multiple colonization events of the body surface of the Florida manatee by diplogastrid nematodes, i.e., *C. manati*, the LT morphospecies and the ST morphospecies that are phylogenetically separate, is very interesting and challenges our thinking about how such an association originated. However, the terrestrial adaptations suggest that this group shares a tolerance for the waste metabolites of other organisms as well as an ability to endure shifts in osmotic pressure. These adaptations may have allowed diplogastrids to exploit a niche within the sloughing dead skin layer of the manatee epidermis while intermittently being exposed to both saltwater and freshwater, i.e., hypothesized to be a preadaptation. In other rhabditid and diplogastrid nematodes, species adapted to nutrient-rich conditions, e.g., dung and decaying carcasses, exhibited extreme arsenic resistance as a preadaptation^24^. The colonization of manatee skin by the three diplogastrid nematodes found in the present study could be another case of habitat-dependent preadaptation, but this will need to be investigated further with manatees and/or dugongs in saltwater/marine environments. Also, further survey work is needed for nematodes in biofouling layers or epidermal substrates of other aquatic animals to confirm the uniqueness of the nematodes reported herein and to search for other interesting symbiotic associations involving the amazing world of roundworms. For example, a recent study of the epibiont meiofauna of loggerhead turtles, *Caretta caretta* L. in Florida found heretofore unreported marine nematode diversity from the carapaces of these seafaring vertebrates and implied a potential phoretic mechanism for large scale movement of benthic meiofauna^20^.

## Materials and methods

### Florida manatee sampling

**Figure 3:**
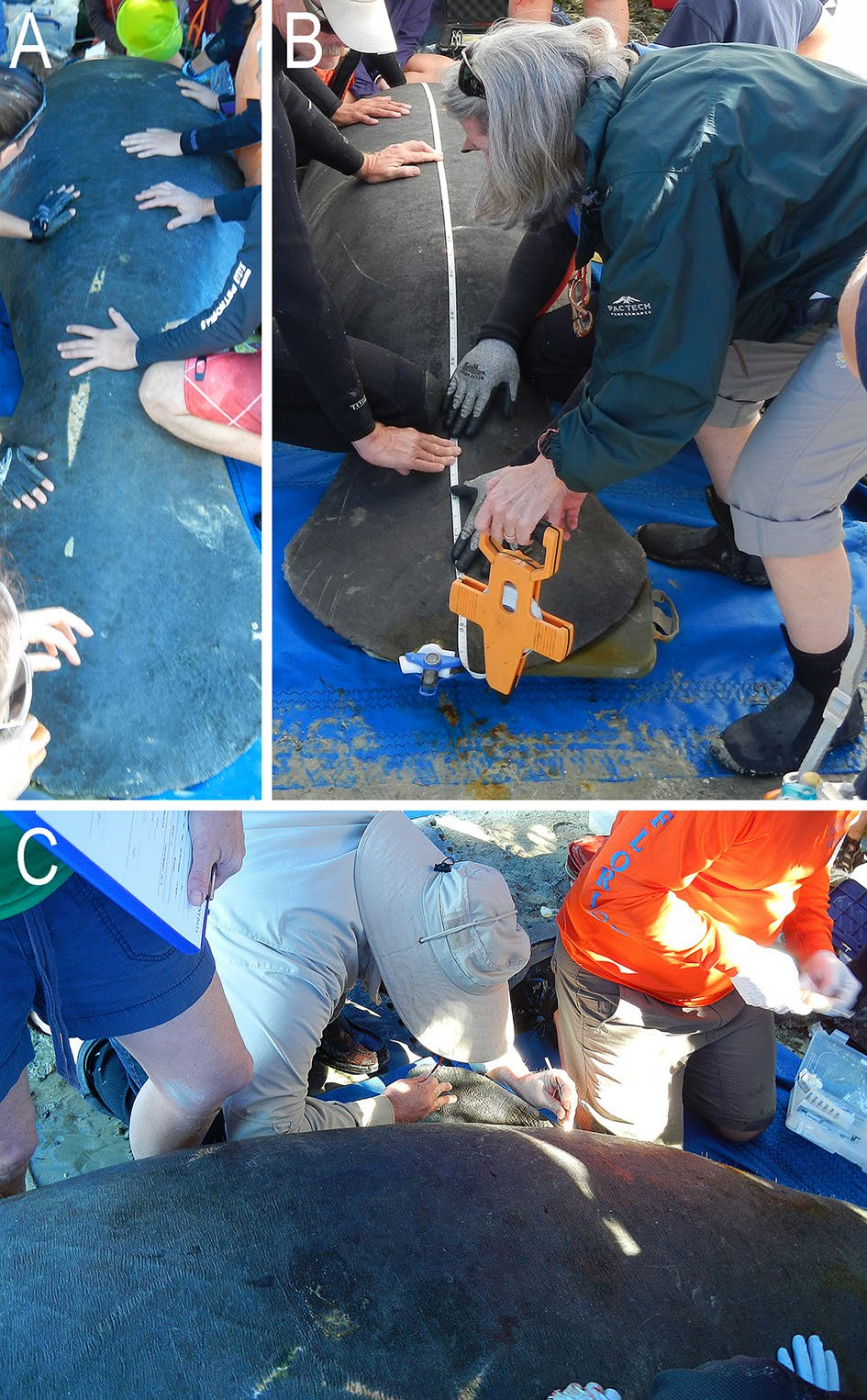
Manatee skin sampling during health assessment. (**A**) Manatee under tent on beach while veterinary personnel perform diagnostics. (**B**) Curvilinear length being measured. (**C**) Skin sample being collected from under the peduncle.

Skin scrapings were collected during the annual Florida Fish and Wildlife Conservation Commission (FWC) health survey of the Florida manatee (*T. m. latirostris*) in the Crystal River National Wildlife Refuge (28°53′28″N, 82°35′50″W) (Fig. 3)^25^. The manatee capturing and research was conducted under the Federal Fish and Wildlife permit number MA791721-5 as well as The Institutional Animal Care and Use Committees (IACUC) permit number USGS/WARC/GNV2019-01. Detailed information about the examined manatees are summarized in Supplementary Table S1. In the first sampling on 3–4 December 2013, each of seven manatees was scraped with a new stainless steel razor blade (2 × 4 cm) over a small area (ca 20 cm^2^) and the residue was transferred to DESS^26^ in a sealed microfuge tube for preservation for subsequent rehydration and morphological observations and DNA amplification attempts. The second sampling occurred on 31 January 2014 and involved six manatees and was done as above except that four samples were deposited directly onto 10% water agar (W/V) plates (100 mm plastic Petri dishes). The third sampling occurred on 7 November 2014 and involved six manatees CCR-14-14 through CCR-14-19 and all samples were scraped onto 10% water agar using several different tools (razor blade, glass slide (2.5 × 7.5 cm), plastic knife and a stainless steel Fisherbrand Spoonula lab spoon [‘spoon’ side = 1.4 × 3.2 cm]) to qualitatively assess harvest efficiency and potential for skin damage during sampling. In addition, due to the danger of handling a large manatee (to the handler and the manatee) and the limited amount of time available for sampling when the manatee is out of water, several samples per manatee were taken at different locations, i.e., dorsal mid-back, dorsal mid-tail, dorsal mid-flank area, and dorsal peduncle fold, to qualitatively assess relative abundance for establishing the subsequent sampling protocols. All three morphospecies of the diplogastrid nematodes found in this study were recovered in similar abundances/frequencies from the different locations sampled. Thus, dorsal mid-tail sampling was used for all subsequent samples because of ease of access and safety.

Sampling was further standardized to involve the Spoonula lab spoon applied nearly parallel to the manatee skin surface using uniform pressure (equivalent to a human back scratch) with consecutive left to right passes being made over an entire 5 × 5 cm sample area on the mid-dorsal region of the tail. When each sample area was completely scraped, or when the spoon was full of collected material, it was transferred to a 10% water agar plate by slicing into the agar at an angle. For samples with large amounts of material, multiple transfers to the same agar plate were done until the entire 5 × 5 cm area had been sampled. Collected sample plates were sealed with a double layered wrap of Parafilm M, placed in Ziploc LDPE (low density polyethylene) plastic bags, stored at ambient temperature and protected from sunlight for the duration of the capture event. On 6 December 2018, 4–9 individual samples were taken from the mid-dorsal tail of each of the seven manatees. On 12 December 2019, 4–6 sequential samples were performed on the same 5 × 5 cm sample area of the seven manatees sampled by gradually increasing the pressure applied while scraping the entire area and each subsequent sample was then transferred to its own agar plate.

### Nematode examination

Sample plates were examined for nematodes under a dissecting microscope or inverted microscope and individually selected for detailed morphotyping using an Olympus BH-2 light microscope with DIC (Differential interference contrast) optics before being photo- or video-micrographed and transferred to nematode digestion buffer^27,28^ for DNA extraction and amplification attempts (see below). All photomicrography was captured through the microscope using a model AM-7023 Dinoeye Eyepiece Camera (AnMo Electronics). Photomicrographs were edited using Adobe Photoshop Elements 9 for figure construction and minor adjustments in brightness and contrast. In addition, each sample plate was observed under the inverted or dissecting microscope to evaluate the morphotype diversity and relative abundance (0, +  = 1–20, ++  = 21–99, and +++  =  > 99)] for each individual manatee within 48 h of collection and rechecked over the next few weeks to assess for survival or any subsequent development. Several attempts were made to transfer nematodes onto different growth media for bacterial (Tryptic Soy Broth [TSB] agar) or fungal (Potato Dextrose agar [PDA]) development and associated nematode reproduction. Attempts were made to optimize the growth media. Water sourced from the manatee sampling location was used to formulate media on some occasions; it was sterilized prior to use. Sterilized water sourced from the same locale was also used to provide irrigation on plates into which channels and pools had been created by excising agar and adding water to fill but not cover the media in the plate. In some cases young individual diplogastrids appeared, presumably hatched from pre-existing eggs, and on at least one occasion an egg was newly laid while the nematode was being observed. Some plates had individual worms still alive up to 5 weeks after sampling, but the diplogastrid populations always steadily declined and were not successfully maintained in culture. There did not appear to be an appreciable difference in success between using ordinary de-ionized water and the water collected at the sampling location. Supplementation of water agar with TSB chunks resulted in more rapid decline compared to water agar alone.

### Molecular characterisation

Nematodes transferred to ISOHAIR digestion buffer were digested at 55 °C for 120 min. followed by an additional three hours at 80 °C and lysates served as the PCR template^27,28^. Because of inconsistent amplifications using single worms, further attempts were made with pooled samples involving 3–7 worms for each morphotype which were ultimately successful.

Sequencing focused on the D2/D3 expansion segments of the large subunit (LSU) ribosomal RNA gene as well as near full-length small subunit (SSU) gene sequences for each of the three different diplogastrid morphospecies that were routinely observed from manatee skin samples. LSU amplification was performed using the primer pair forward D2a (5′-ACAAGTACCGTGAGGGAAAGTTG-3′) and reverse D3b (5′-TGCGAAGGAACCAGCTACTA-3′)^29^. Each 25 μL total volume polymerase chain reaction (PCR) was formulated using Genessee Sci brand Apex Master Mix taq polymerase (Genesee Sci Inc., San Diego CA, USA) in accordance with the manufacturer’s protocol, inclusive of 1 μL of template DNA per reaction. Thermal cycling was conducted in the following steps using a Veriti VeriFlex Thermal Cycler (Applied Biosystems via Thermo Fisher Scientific): an initial heating to 95 °C for 5 min, then 35 cycles each consisting of denaturation at 95 °C for 1 min, annealing at 55 °C for 1 min, and extension at 72 °C for 1 min. A final extension step was performed at 72 °C for 10 min. PCR products were purified using ExoSap-IT (Affymetrix Inc., Santa Clara CA, USA) and sent to EUROFINS USA for final sequencing from the purified PCR products. Small subunit (SSU) ribosomal RNA gene sequence products were also amplified. SSU PCR was conducted using the same thermal cycling parameters described above with two sets of primer pairs. The ‘front’ primer pair for SSU were forward SSU988F (5′-CTCAAAGATTAAGCCATGC-3′ and reverse SSU1912R (5′-CTCAAAGATTAAGCCATGC-3′), while the ‘rear’ primer pair were forward SSU1813F (5′-CTGCGTGAGAGGTGAAAT-3′ and reverse SSU2646R (5′- GCTACCTTGTTACGACTTTT-3′)^30^. LSU and SSU sequences were aligned and reconciled to consensus using Geneious 8.1.3 (https://www.geneious.com) software.

LSU and SSU sequence data for each of the three representative diplogastrid morphospecies was uploaded to The National Center for Biotechnology Information (NCBI) GenBank as accession numbers MT160762 (*C. manati* LSU), MT160758 (*C. manati* SSU), MT160763 (LT LSU), MT160759 (LT SSU), MT160764 (ST LSU) and MT160760 (ST SSU). These sequences were compared with those deposited in the database using BLAST homology search program (https://blast.ncbi.nlm.nih.gov/Blast.cgi?PROGRAM=blastn&PAGE_TYPE=BlastSearch&LINK_LOC=blasthome). The isolates for tree construction were selected according to the BLAST results and sequences from previous publications^8,15,29–31^. The compared sequences are summarized in Table S2. *Rhabditoides inermis* which is the basal to Rhabditidae + Diplogastridae clade^30^ served as an outgroup species.

Both SSU and D2–D3 LSU sequences were used for a combined phylogenetic analysis. The compared sequences were aligned using MAFFT^32,33^ (available online at http://align.bmr.kyushu-u.ac.jp/mafft/software/), and the substitution model and parameters were determined by MEGA X software^34^ using Akaike information criterion (AIC) model selection. The phylogenetic relationships of the three diplogastrid morphotypes were inferred with MrBayes 3.2^35,36^ and according to Kanzaki et al.^37^. Finalized results were used for tree construction as described by Ye et al.^29^.

## Supplementary Information

Supplementary Information.
