# Supplementary material for: Nematode epibionts on skin of the Florida manatee, Trichechus manatus latirostris
Source: Sci Rep. 2021 Jan 13;11:1211. doi: 10.1038/s41598-020-79879-7 (PMC7806751; doi:10.1038/s41598-020-79879-7)
Supplement: Supplementary file 1 — Supplementary Information. [file 41598_2020_79879_MOESM1_ESM.pptx]

## Slide 1
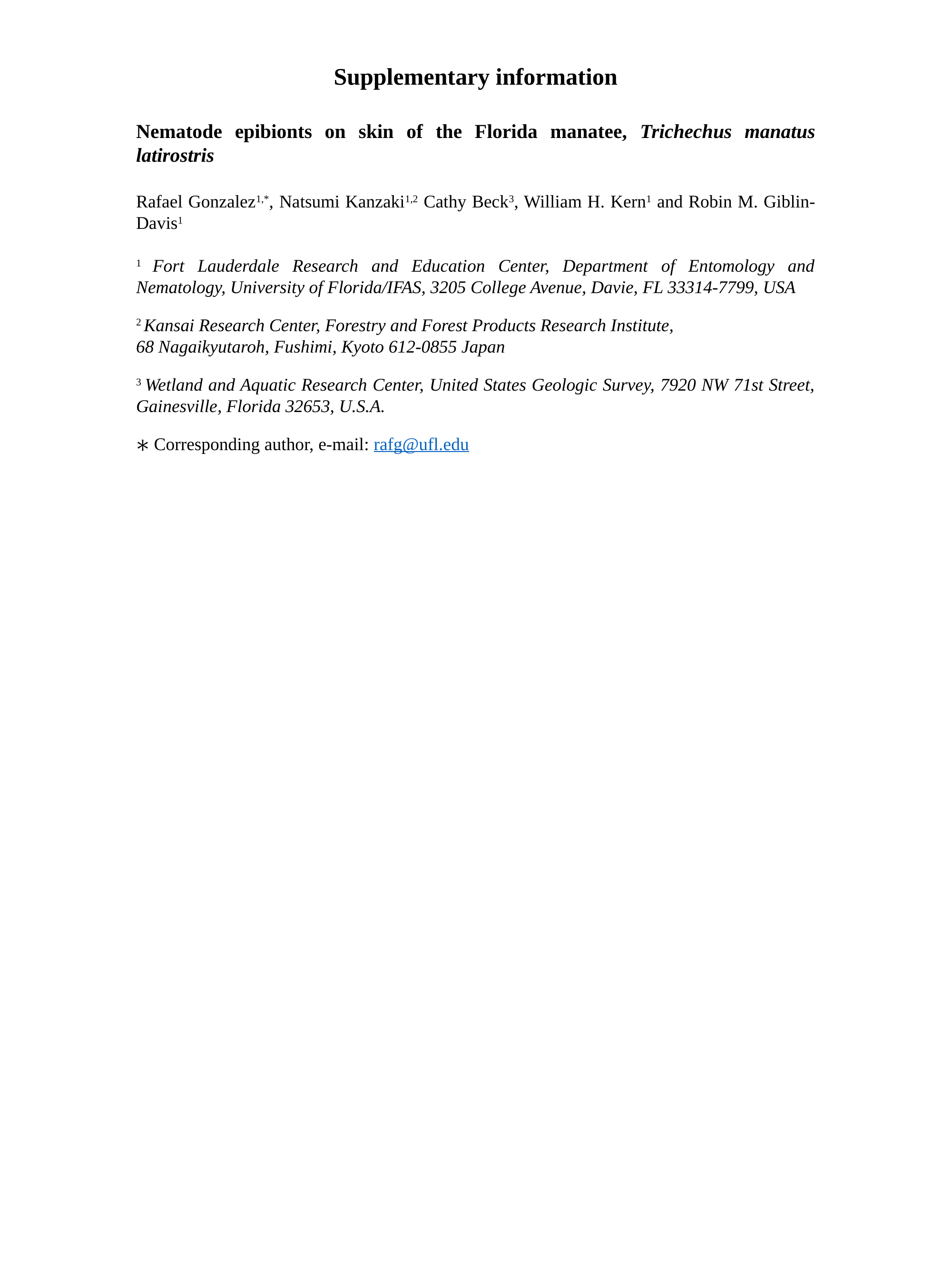

Supplementary information
Nematode epibionts on skin of the Florida manatee, Trichechus manatus latirostris
Rafael Gonzalez1,*, Natsumi Kanzaki1,2 Cathy Beck3, William H. Kern1 and Robin M. Giblin-Davis1
1 Fort Lauderdale Research and Education Center, Department of Entomology and Nematology, University of Florida/IFAS, 3205 College Avenue, Davie, FL 33314-7799, USA
2 Kansai Research Center, Forestry and Forest Products Research Institute,
68 Nagaikyutaroh, Fushimi, Kyoto 612-0855 Japan
3 Wetland and Aquatic Research Center, United States Geologic Survey, 7920 NW 71st Street, Gainesville, Florida 32653, U.S.A.
∗ Corresponding author, e-mail: rafg@ufl.edu

## Slide 2
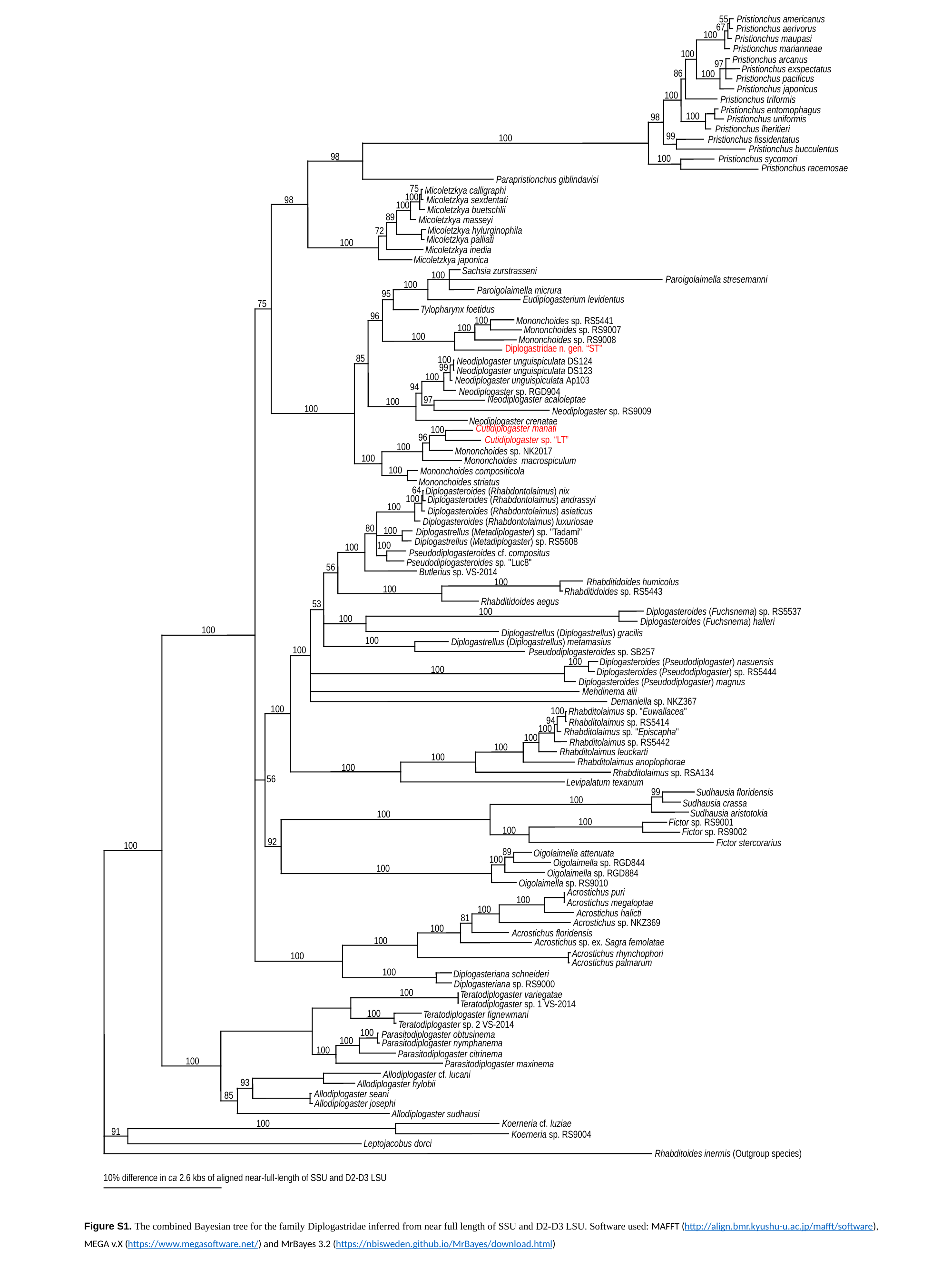

55
Pristionchus americanus
67
Pristionchus aerivorus
100
Pristionchus maupasi
Pristionchus marianneae
100
Pristionchus arcanus
97
Pristionchus exspectatus
86
100
Pristionchus pacificus
Pristionchus japonicus
100
Pristionchus triformis
Pristionchus entomophagus
100
98
Pristionchus uniformis
Pristionchus lheritieri
99
100
Pristionchus fissidentatus
Pristionchus bucculentus
98
100
Pristionchus sycomori
Pristionchus racemosae
Parapristionchus giblindavisi
75
Micoletzkya calligraphi
100
98
Micoletzkya sexdentati
100
Micoletzkya buetschlii
89
Micoletzkya masseyi
Micoletzkya hylurginophila
72
Micoletzkya palliati
100
Micoletzkya inedia
Micoletzkya japonica
Sachsia zurstrasseni
100
Paroigolaimella stresemanni
100
Paroigolaimella micrura
95
Eudiplogasterium levidentus
75
Tylopharynx foetidus
96
100
Mononchoides sp. RS5441
100
Mononchoides sp. RS9007
100
Mononchoides sp. RS9008
Diplogastridae n. gen. “ST”
85
100
Neodiplogaster unguispiculata DS124
99
Neodiplogaster unguispiculata DS123
100
Neodiplogaster unguispiculata Ap103
94
Neodiplogaster sp. RGD904
Neodiplogaster acaloleptae
97
100
100
Neodiplogaster sp. RS9009
Neodiplogaster crenatae
Cutidiplogaster manati
100
96
Cutidiplogaster sp. “LT”
100
Mononchoides sp. NK2017
100
Mononchoides macrospiculum
100
Mononchoides compositicola
Mononchoides striatus
64
Diplogasteroides (Rhabdontolaimus) nix
100
Diplogasteroides (Rhabdontolaimus) andrassyi
100
Diplogasteroides (Rhabdontolaimus) asiaticus
Diplogasteroides (Rhabdontolaimus) luxuriosae
80
100
Diplogastrellus (Metadiplogaster) sp. "Tadami"
Diplogastrellus (Metadiplogaster) sp. RS5608
100
100
Pseudodiplogasteroides cf. compositus
Pseudodiplogasteroides sp. "Luc8"
56
Butlerius sp. VS-2014
100
Rhabditidoides humicolus
100
Rhabditidoides sp. RS5443
Rhabditidoides aegus
53
100
Diplogasteroides (Fuchsnema) sp. RS5537
100
Diplogasteroides (Fuchsnema) halleri
100
Diplogastrellus (Diplogastrellus) gracilis
100
Diplogastrellus (Diplogastrellus) metamasius
100
Pseudodiplogasteroides sp. SB257
100
Diplogasteroides (Pseudodiplogaster) nasuensis
100
Diplogasteroides (Pseudodiplogaster) sp. RS5444
Diplogasteroides (Pseudodiplogaster) magnus
Mehdinema alii
Demaniella sp. NKZ367
100
100
Rhabditolaimus sp. "Euwallacea"
94
Rhabditolaimus sp. RS5414
100
Rhabditolaimus sp. "Episcapha"
100
Rhabditolaimus sp. RS5442
100
Rhabditolaimus leuckarti
100
Rhabditolaimus anoplophorae
100
Rhabditolaimus sp. RSA134
56
Levipalatum texanum
99
Sudhausia floridensis
100
Sudhausia crassa
Sudhausia aristotokia
100
100
Fictor sp. RS9001
100
Fictor sp. RS9002
92
Fictor stercorarius
100
89
Oigolaimella attenuata
100
Oigolaimella sp. RGD844
100
Oigolaimella sp. RGD884
Oigolaimella sp. RS9010
Acrostichus puri
100
Acrostichus megaloptae
100
Acrostichus halicti
81
Acrostichus sp. NKZ369
100
Acrostichus floridensis
100
Acrostichus sp. ex. Sagra femolatae
Acrostichus rhynchophori
100
Acrostichus palmarum
100
Diplogasteriana schneideri
Diplogasteriana sp. RS9000
100
Teratodiplogaster variegatae
Teratodiplogaster sp. 1 VS-2014
100
Teratodiplogaster fignewmani
Teratodiplogaster sp. 2 VS-2014
100
Parasitodiplogaster obtusinema
100
Parasitodiplogaster nymphanema
100
Parasitodiplogaster citrinema
100
Parasitodiplogaster maxinema
Allodiplogaster cf. lucani
93
Allodiplogaster hylobii
Allodiplogaster seani
85
Allodiplogaster josephi
Allodiplogaster sudhausi
100
Koerneria cf. luziae
91
Koerneria sp. RS9004
Leptojacobus dorci
Rhabditoides inermis (Outgroup species)
10% difference in ca 2.6 kbs of aligned near-full-length of SSU and D2-D3 LSU
Figure S1. The combined Bayesian tree for the family Diplogastridae inferred from near full length of SSU and D2-D3 LSU. Software used: MAFFT (http://align.bmr.kyushu-u.ac.jp/mafft/software), MEGA v.X (https://www.megasoftware.net/) and MrBayes 3.2 (https://nbisweden.github.io/MrBayes/download.html)

## Slide 3
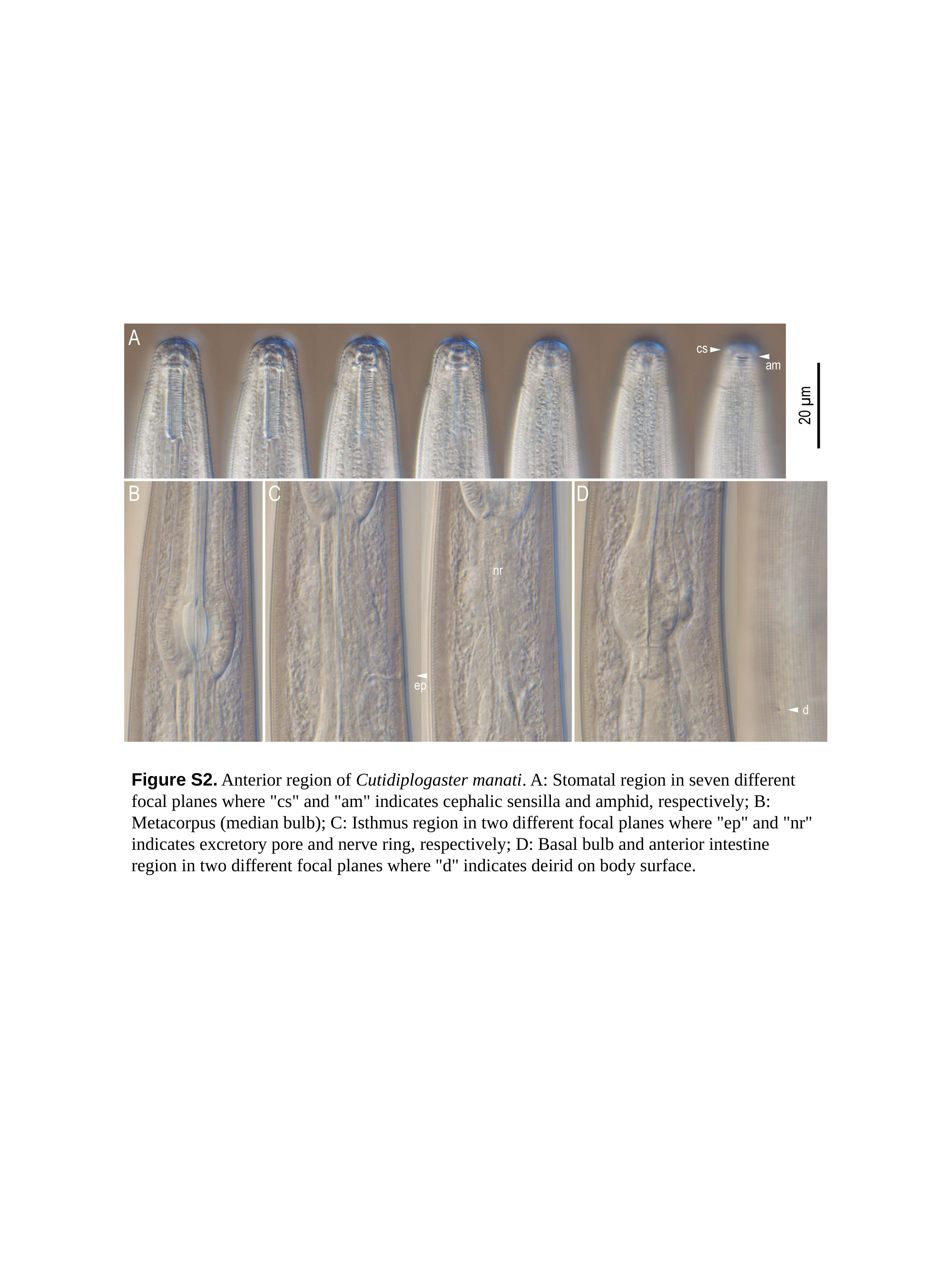

Figure S2. Anterior region of Cutidiplogaster manati. A: Stomatal region in seven different focal planes where "cs" and "am" indicates cephalic sensilla and amphid, respectively; B: Metacorpus (median bulb); C: Isthmus region in two different focal planes where "ep" and "nr" indicates excretory pore and nerve ring, respectively; D: Basal bulb and anterior intestine region in two different focal planes where "d" indicates deirid on body surface.

## Slide 4
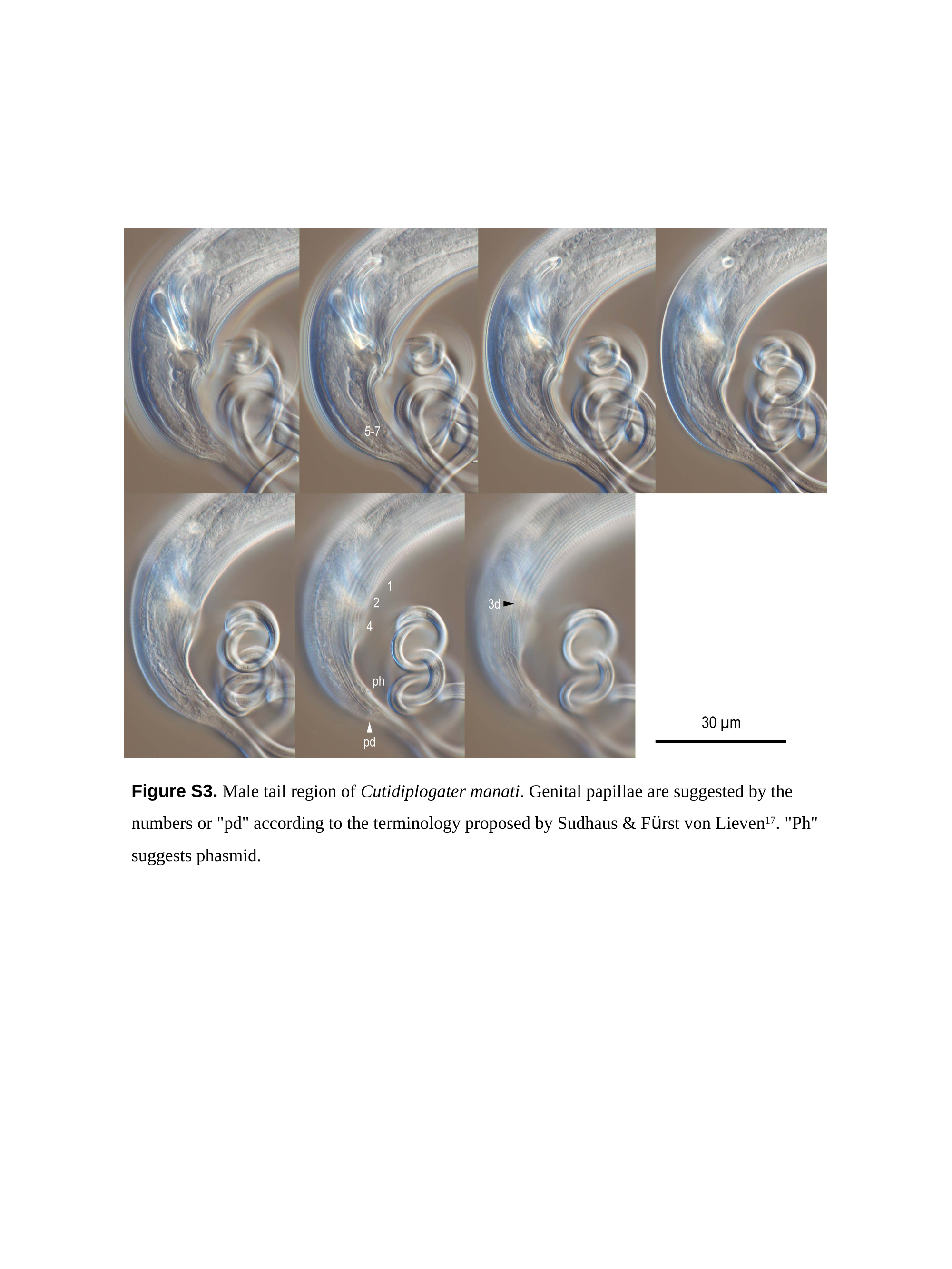

Figure S3. Male tail region of Cutidiplogater manati. Genital papillae are suggested by the numbers or "pd" according to the terminology proposed by Sudhaus & Fürst von Lieven17. "Ph" suggests phasmid.

## Slide 5
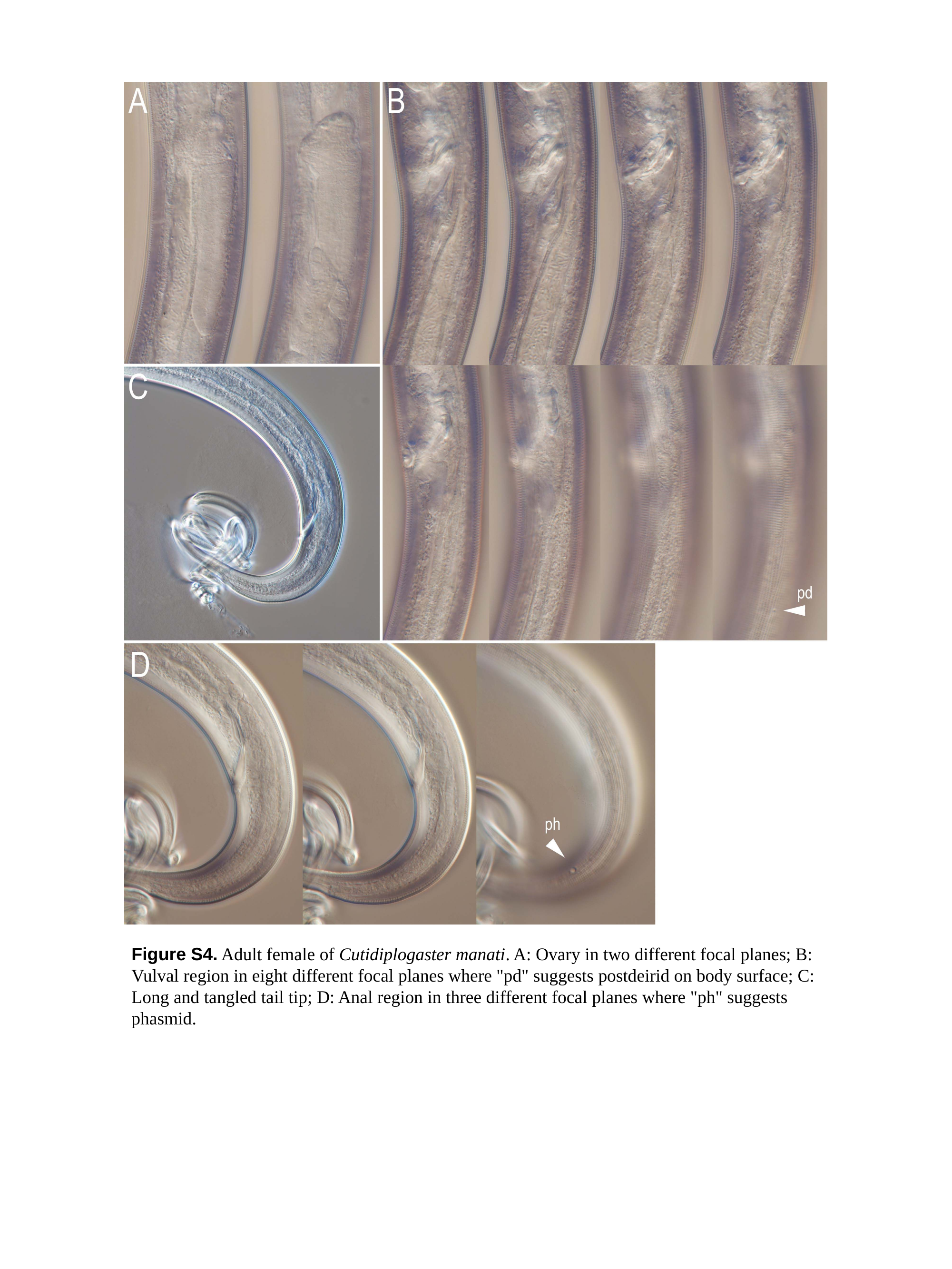

Figure S4. Adult female of Cutidiplogaster manati. A: Ovary in two different focal planes; B: Vulval region in eight different focal planes where "pd" suggests postdeirid on body surface; C: Long and tangled tail tip; D: Anal region in three different focal planes where "ph" suggests phasmid.

## Slide 6
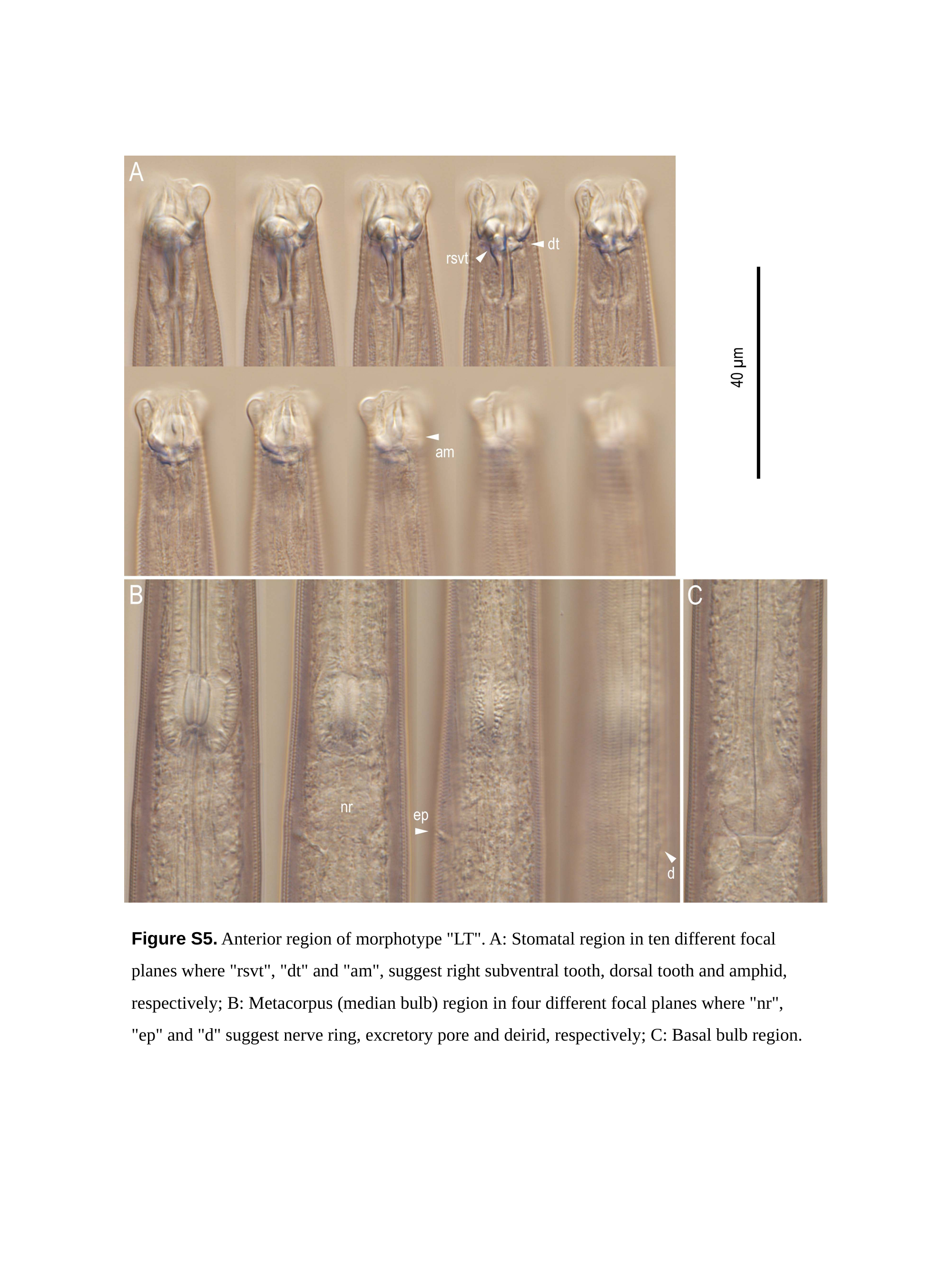

Figure S5. Anterior region of morphotype "LT". A: Stomatal region in ten different focal planes where "rsvt", "dt" and "am", suggest right subventral tooth, dorsal tooth and amphid, respectively; B: Metacorpus (median bulb) region in four different focal planes where "nr", "ep" and "d" suggest nerve ring, excretory pore and deirid, respectively; C: Basal bulb region.

## Slide 7
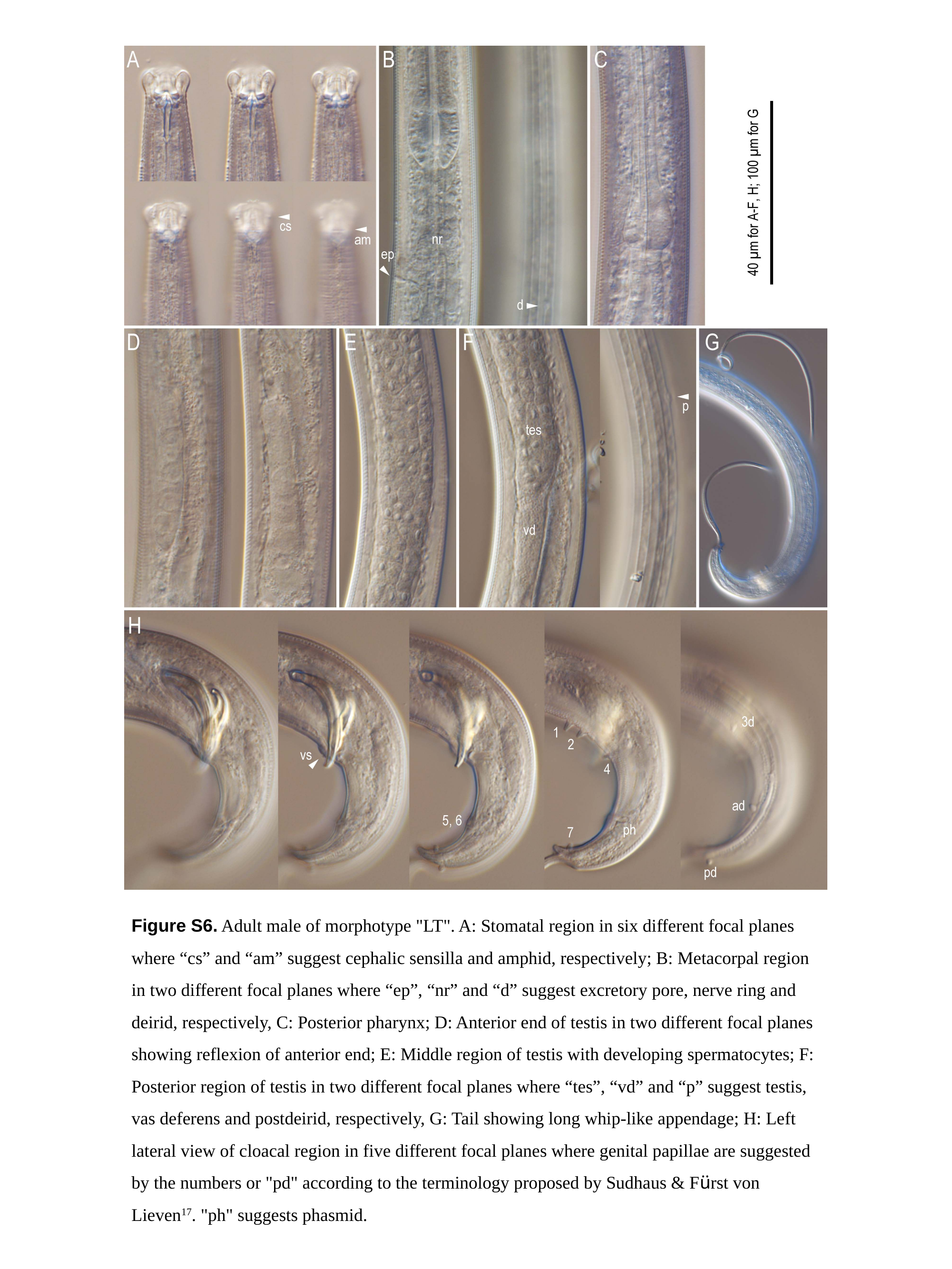

Figure S6. Adult male of morphotype "LT". A: Stomatal region in six different focal planes where “cs” and “am” suggest cephalic sensilla and amphid, respectively; B: Metacorpal region in two different focal planes where “ep”, “nr” and “d” suggest excretory pore, nerve ring and deirid, respectively, C: Posterior pharynx; D: Anterior end of testis in two different focal planes showing reflexion of anterior end; E: Middle region of testis with developing spermatocytes; F: Posterior region of testis in two different focal planes where “tes”, “vd” and “p” suggest testis, vas deferens and postdeirid, respectively, G: Tail showing long whip-like appendage; H: Left lateral view of cloacal region in five different focal planes where genital papillae are suggested by the numbers or "pd" according to the terminology proposed by Sudhaus & Fürst von Lieven17. "ph" suggests phasmid.

## Slide 8
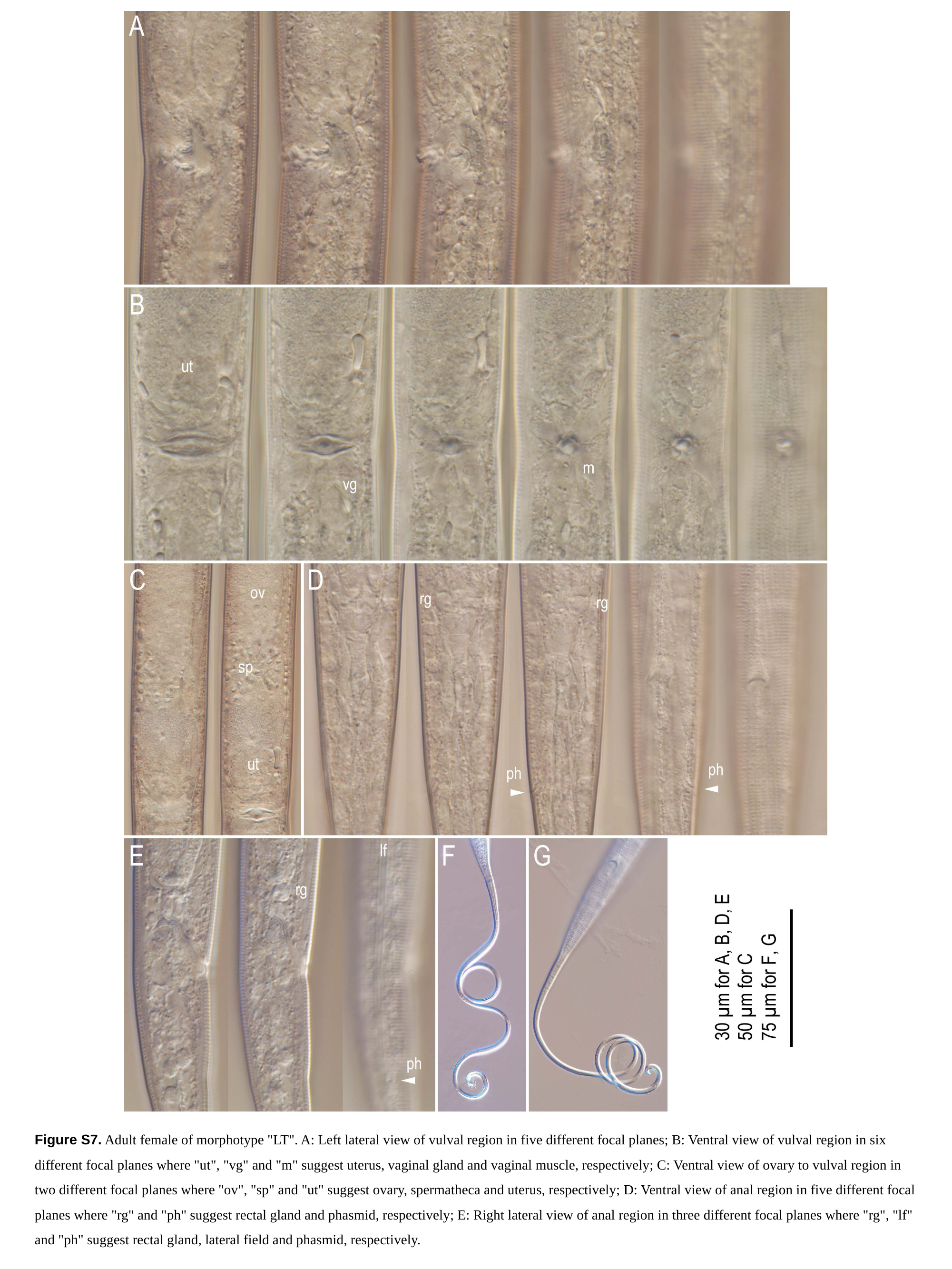

Figure S7. Adult female of morphotype "LT". A: Left lateral view of vulval region in five different focal planes; B: Ventral view of vulval region in six different focal planes where "ut", "vg" and "m" suggest uterus, vaginal gland and vaginal muscle, respectively; C: Ventral view of ovary to vulval region in two different focal planes where "ov", "sp" and "ut" suggest ovary, spermatheca and uterus, respectively; D: Ventral view of anal region in five different focal planes where "rg" and "ph" suggest rectal gland and phasmid, respectively; E: Right lateral view of anal region in three different focal planes where "rg", "lf" and "ph" suggest rectal gland, lateral field and phasmid, respectively.

## Slide 9
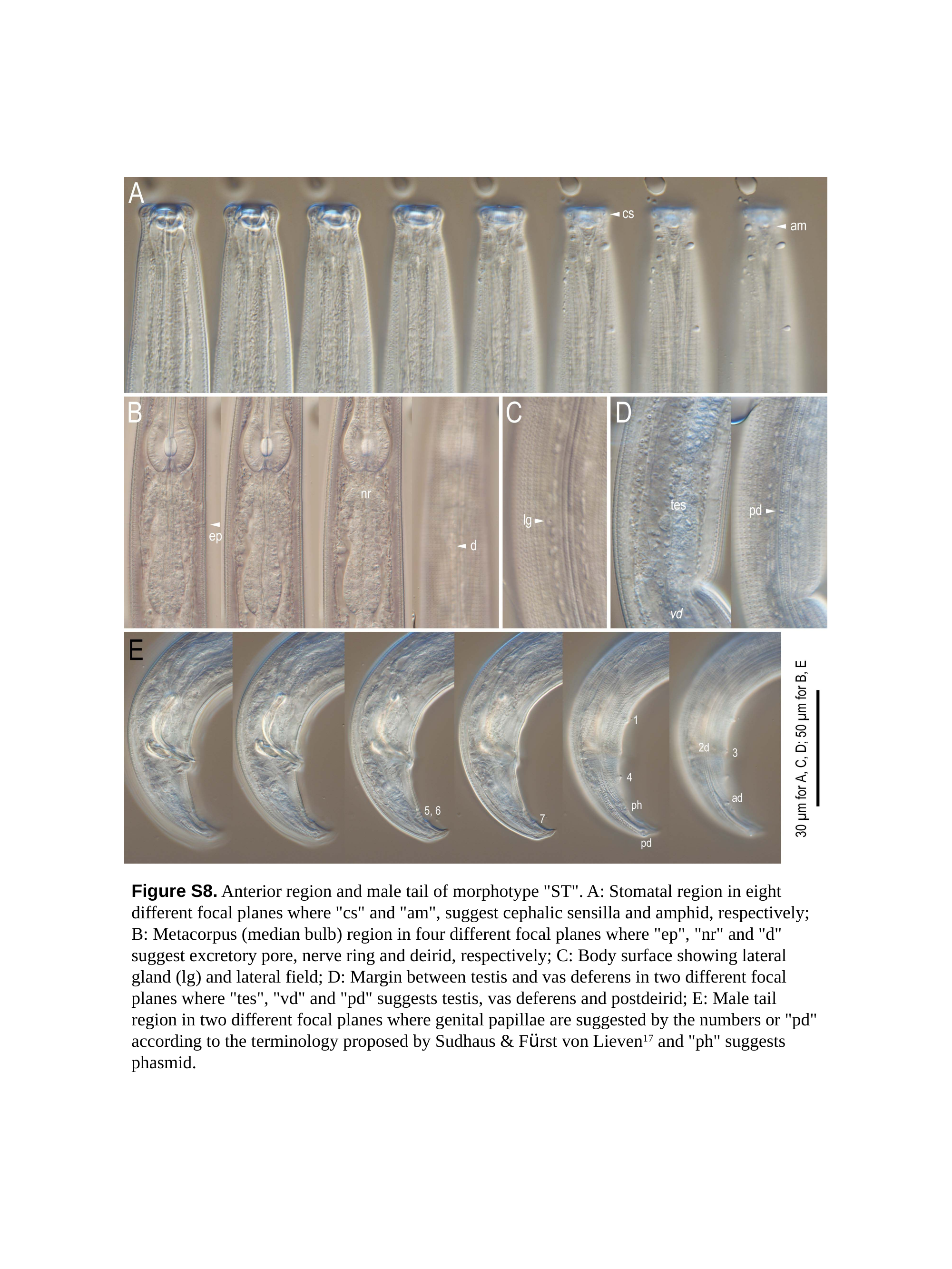

Figure S8. Anterior region and male tail of morphotype "ST". A: Stomatal region in eight different focal planes where "cs" and "am", suggest cephalic sensilla and amphid, respectively; B: Metacorpus (median bulb) region in four different focal planes where "ep", "nr" and "d" suggest excretory pore, nerve ring and deirid, respectively; C: Body surface showing lateral gland (lg) and lateral field; D: Margin between testis and vas deferens in two different focal planes where "tes", "vd" and "pd" suggests testis, vas deferens and postdeirid; E: Male tail region in two different focal planes where genital papillae are suggested by the numbers or "pd" according to the terminology proposed by Sudhaus & Fürst von Lieven17 and "ph" suggests phasmid.

## Slide 10
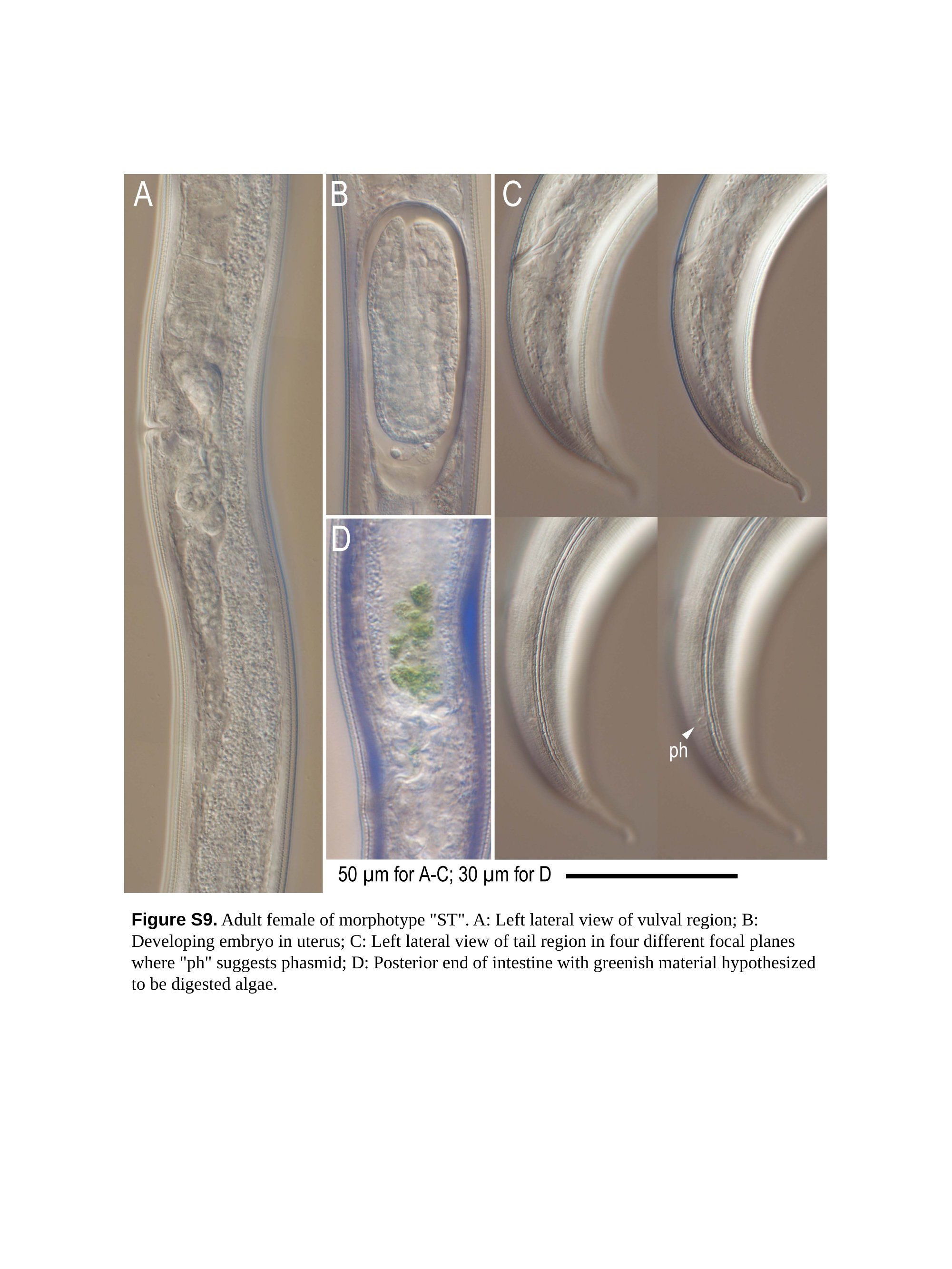

Figure S9. Adult female of morphotype "ST". A: Left lateral view of vulval region; B: Developing embryo in uterus; C: Left lateral view of tail region in four different focal planes where "ph" suggests phasmid; D: Posterior end of intestine with greenish material hypothesized to be digested algae.
